# Quantitative trait loci (QTL) for low temperature tolerance at the young microspore stage in rice (*Oryza sativa* L.) in Australian breeding material

**DOI:** 10.1270/jsbbs.21096

**Published:** 2022-06-16

**Authors:** Christopher Proud, Bradley Campbell, Zuziana Susanti, Shu Fukai, Ian Godwin, Ben Ovenden, Peter Snell, Jaquie Mitchell

**Affiliations:** 1 The University of Queensland, School of Agriculture and Food Sciences, St Lucia, Queensland 4072, Australia; 2 Indonesian Centre for Rice Research, Agency for Agricultural Research and Development, Subang, West-Java, Indonesia; 3 Queensland Alliance for Agriculture and Food Innovation, The University of Queensland, St Lucia, Queensland 4072, Australia; 4 Department of Primary Industries, Yanco Agricultural Institute, Yanco, NSW 2703, Australia

**Keywords:** young microspore stage, low temperature tolerance, spikelet fertility, temperate rice

## Abstract

Low temperatures at the young microspore stage (YMS) decreases spikelet fertility and is a major limiting factor to rice production in temperate Australia. Low temperature tolerance is a difficult trait to phenotype, hence there is a strong desire for the identification of quantitative trait loci (QTL) for their use in marker-assisted selection (MAS). Association mapping was used in several breeding populations with a known source of low temperature tolerance, Norin PL8, to identify QTL for low temperature tolerance. A novel QTL for spikelet fertility was identified on chromosome 6, *qYMCT6.1*, in which the Australian variety, Kyeema, was the donor for increased fertility. Additional five genomics regions were identified that co-located with previously reported QTL, two of which have been previously cloned. Additionally, for the first time a QTL for spikelet fertility *qYMCT10.1*, has been shown to co-locate with the number of dehisced anthers *qYMCTF10.1* which increases the shedding of pollen from the anthers. This study revealed one new QTL for low temperature tolerance at YMS in temperate japonica germplasm and identified an additional five previously reported. These QTL will be utilised for MAS in the Australian rice breeding program and may have merit for temperate breeding programs globally.

## Introduction

Rice (*Oryza sativa* L.) is one of the principal staple foods feeding humanity and a key crop in the maintenance of food security throughout much of Asia. Productivity is limited in high elevation and/or high latitude growing regions such as China, Japan, Korea and Australia, due to low temperature events at the reproductive stage which can cause large reductions in yield. The most sensitive stage for low temperature injury in rice, particularly in Australia, is the booting stage, specifically the young pollen microspore stage, which occurs approximately 10–12 days prior to heading (Satake and Hayase 1970). Temperatures considered detrimental to pollen grain formation sit in the range of 15 to 19°C and result in reduced spikelet fertility (Satake 1976, Shimono *et al.* 2002, 2011). The probability of an Australian rice crop experiencing mean night temperatures less than 15°C for a period of 10 days is as high as 80% in November and 50% in late February when flowering commonly occurs (Farrell *et al.* 2001).

To overcome this low temperature constraint, rice genetic resources displaying tolerance to low temperature have been identified and crossed with elite cultivars to create tolerant varieties (Ye *et al.* 2010). However, delivering reliable genetic gains for the trait is a difficult undertaking given the complexity of developing reliable phenotyping systems, particularly in the field. Consequently, there is a desire for the identification of quantitative trait loci (QTL) and associated markers for use in marker-assisted selection (MAS) to improve the breeding efficiencies for low temperature tolerance. There are two important steps that are necessary for the potential deployment of the identified QTL; firstly, validation of the QTL in multiple backgrounds and secondly, the development of appropriate markers to target the QTL. The validation of QTL in multiple backgrounds is often overlooked in deployment of markers in MAS which can lead to poor outcomes when using MAS (Cobb *et al.* 2019).

QTL for low temperature tolerance at the booting stage have been mapped on all chromosomes. To date, there have been six cloned genes identified for low temperature tolerance at the reproductive stage: *Ctb-1*, *qCTB4a*, *qCTB2* (*qCTB4b*), *ltt1*, *bZIP73* and *qPSR10* (Li *et al.* 2021, Liu *et al.* 2019, Saito *et al.* 2010, Xiao *et al.* 2018, Xu *et al.* 2020, Zhang *et al.* 2017). The results therein demonstrate multiple pathways for tolerance with *ltt1* maintaining tapetum degradation by activation of systems metabolising reactive oxygen species (Xu *et al.* 2020), whereas *qCTB4a* and *qCTB2* mediate adenosine triphosphate synthesis and sterol metabolism, respectively (Li *et al.* 2021). *bZIP73* is a transcription factor which is involved in the modulation of abscisic acid and reactive oxygen species (Liu *et al.* 2019). The underlying molecular mechanisms of *Ctb-1* and *qPSR10* is not well understood. However, the end result is increased male fertility leading to increased probability of fertilisation and thus increased fertility with four of six genes previously reported to improve pollen viability and/or anther length. The relationship between spikelet fertility and floral traits particularly pollen in anther has been well demonstrated in the literature (Farrell *et al.* 2006a, Gunawardena *et al.* 2003, Susanti *et al.* 2019). More recently, Susanti *et al.* (2019) demonstrated that the amount of anther dehiscence contributed significantly to low temperature tolerance and spikelet fertility. Despite having long anthers, Australian rice varieties have low pollen loads compared with other *japonica* contemporaries (Farrell *et al.* 2006a, 2006b).

All of the genes previously cloned, with the exception of *ltt1*, as well as other QTL identified for low temperature tolerance have only had small to moderate effect which highlight the highly polygenic nature of low temperature tolerance and as such, pyramiding multiple QTL is a necessity to achieve a high level of tolerance. However, any QTL and associated marker needs to demonstrate its utilitarian value to the breeding program. The genetic material that has been reported in this paper emanates directly from within the Australian breeding program. As such the material phenotyped are not ideal populations for QTL mapping studies, however they are directly relevant to the Australian breeding program.

In this paper we used several backcross populations utilising Kyeema and Norin PL8, and subsequent multibackground populations derived from Kyeema//Kyeema/NorinPL8 to identify QTL for potential marker-assisted selection. In addition, the consistency of the genetic control between spikelet fertility and floral traits was explored.

## Materials and Methods

### Populations

The populations used in this study were derived from Australian aromatic long grain, low-temperature sensitive parent *Oryza sativa* L. ‘Kyeema’, and the Japanese cold tolerant donor parent *Oryza sativa* L. ‘Norin PL8’. Norin PL8 is a semi-dwarf short grain previously characterised for low temperature tolerance (Saito *et al.* 1995). Norin PL8 has previously had two closely-linked QTL identified for cold tolerance, *Ctb-1* and *Ctb-2*, one of which has subsequently been cloned (Saito *et al.* 2001, 2010).

This first population consisted of 117 F6 lines, Kyeema//Kyeema/Norin PL8 (KKN), a breeding population developed through single seed descent from BC1F3 head selections. A backcrossed population was employed as it was more likely to generate progeny of merit to the quality requirements of a soft-cooking low temperature tolerant long grain. In brief, two F3 low temperature tolerant lines identified in the F2 low temperature nursery of Kyeema/Norin PL8 were backcrossed to Kyeema from which 2 F1 populations (YC08258 and YC08259) were selfed, and the F2 populations were screened in a low temperature nursery in the summer of 2011/2012. Two tolerant individuals were identified in YC08258 from 55 panicles and 7 individuals from 69 panicles in YC08259. Approximately 15 F3s were expedited to the F6, from which 117 lines were harvested.

The material disseminating from the first KKN population while containing a high-level of low temperature tolerance was far from desirable from a commercial perspective and therefore additional crosses were made with KKN derived material and temperate long-grain varieties. These crosses were grouped into two, backcrossed and multiple-background populations, that were both subsequently evaluated.

The second population group consisted of two double backcross populations, YC12147 (KKN/Kyeema) and YC12157 (Kyeema/KKN), consisting of 170 and 140 lines, respectively, which were produced by single seed descent from the F2 population to the F5. The male and female parent of YC12147 and YC12157 were F3s from 2415 and 2476 from the KKN population, respectively.

The third group consisted of multiple-background populations consisted of KKN F3 individuals crossed with *Oryza sativa* L. ‘Topaz’, ‘Doongara’ and ‘YRL126’ which are all Australian long grain varieties and/or advanced breeding lines. The Topaz/KKN (490) were from three individual crossing events derived from three F3 KKN families. YRL126/KKN (97) was from two separate crosses and Doongara/KKN a single cross (40). YRL126//Topaz/KKN was derived from the cross YRL126/YC10416 (47), while YC10416 was from a cross between Topaz and an F1 of YC08258. All lines were F6 with no prior selection and in total the population consisted of 664 lines of which 240 were phenotyped.

### Genotyping

All lines were genotyped using the Diversity Arrays Technology (DArT) genotype-by-sequencing platform, DArTseq, using the method previously described by Courtois *et al.* (2013). Briefly, methylation sensitive restrictive enzymes were used for complexity reduction of genomic DNA before Illumina short-read sequencing. The resulting sequences were aligned to Oryza sativa v7.0 reference genome (https://jgi.doe.gov/) to identify single nucleotide polymorphisms (SNPs). The physical map position was used to denote the position of the SNP. SNP filtering was completed as per Vinarao *et al.* (2021) with the exception of imputation achieved with random forest imputation of 500 trees with the R package ‘missForest v1.4’ (Stekhoven and Bühlmann 2011) and filtering based on a minor allele frequency of 0.05.

Linkage disequilibrium (LD) was measured as the squared allelic correlation between pairs of markers on a chromosome (r^2^) using the R package ‘LDcorSV’ (Mangin *et al.* 2012). Firstly, markers that had an allele frequency below 0.1 were discarded, then marker intervals were discretised into 10 kb intervals, and the median r^2^ was used as the estimate for each interval. Intervals were only included if there were more than 20 comparisons within the interval. Pairwise LD r^2^ estimates were plotted against the corresponding pairwise genetic distances between markers and a second degree locally weighted polynomial regression (LOESS) curve was fitted to the scatterplot (Cleveland 1978).

### Phenotyping method

All three population groups were grown in the two set phenotyping experiments following the method of Mitchell *et al.* (2016). Experiments were conducted across two temperature-controlled glasshouse rooms at the University of Queensland, St Lucia, Queensland, Australia. These glasshouse rooms were located next to each other, and other than temperature, growing conditions were identical. Briefly, the two-set phenotyping method relies on two plantings 17–18 days apart in warm (22/28°C, night/day) controlled temperature conditions. On heading of the 1st plant for each line in the first sown set, the corresponding line in set 2 (YMS exposure) was transferred to low temperatures (15/21°C night/day) for 14 days before being returned to warm temperatures until maturity. The principle of the phenotyping method is that the period between heading and the young microspore stage is approximately 14 days prior to heading under ideal conditions, and as such heading of set 1 is indicative of 3–4 days prior to YMS in set 2, and as such set 2 is exposed at the most sensitive period of plant development.

The cultural details of the experiments followed the protocols established in Susanti *et al.* (2019) except where stated. Heading was defined as the initial protrusion of the terminal spikelet from the sheath on the main stem. Spikelet fertility on the main stem panicle was used to assess the low-temperature tolerance or susceptibility of a line. Percentage spikelet fertility were calculated for each entry in each experiment. Spikelet fertility was calculated as filled spikelet number/total spikelet number (%), where total spikelet number included filled and unfilled spikelet number.

Spikelet fertility under warm conditions was observed in Set1 or the preceding generation to ensure spikelet fertility was primarily influenced by low temperature rather than underlying genetics that may also exist and be expressed under ‘ideal’ conditions. In all cases the populations did not show excessive spikelet sterility, with spikelet fertility greater than 80% in warm conditions for all genotypes.

### Experiment 1: KKN population

The phenotyping of spikelet fertility and floral traits of the KKN population for Experiment 1 was previously discussed in Susanti *et al.* (2019). In brief, the two sets, planted 18 days apart, consisted of 117 lines of the population, parents (Kyeema and Norin PL8) and Sherpa all of which had three replications, and were arranged in the completely randomised design. In addition to spikelet fertility, the experiment also determined the number of pollen in anther, the number of dehisced anthers, dehiscence length and number of pollen on stigma. The first set was planted on 20 March 2014.

### Experiment 2: Double backcrossed populations

Experiment 2 consisted of 132 (YC12147) and 114 (YC12157) lines which were phenotyped for low temperature tolerance at YMS, following the protocol described above. The parents plus 17 checks and breeding lines which are not discussed herein were included in the phenotyping. The first set was designed in a row-column augmented block design using the R package ‘DiGGer’ (Coombes 2009) in which YC12147 and YC12157 were replicated once, eight main checks replicated 7 times and the remaining twice.

In the second set, nine lines of the backcross populations were replicated the once, while all remainder had two replicates. The parents and Sherpa had 5 replications and the remaining checks had 3. In the warm room, a partially-replicated AR1xAR1 row-column design was used across two tubs where the tubs were considered a blocking structure. The first set was planted on the 24 September 2018 and the second set 17 days later. Lines with more than one replication in the first set were moved when more than half the number had headed.

### Experiment 3: Multiple backgrounds

Two-hundred and forty lines from the multiple background population groups as well as 89 checks and breeding lines (as part of a regular evaluation program) were included in the phenotyping experiment. Of the 664 lines, 38 lines from Doongara/KKN and YRL126//Topaz/KKN were included. Thirty-five of YRL126/KKN were selected at random. Of the Topaz/KKN, 20 lines were selected based on having no tolerant allele from Experiments 1 and 2, and the remainder were selected at random. The first set was arranged in an augmented design as per Experiment 2. The second set had three replications across three tubs and were arranged in a spatially-adjusted block design for the warm room. The cultural details follow that as described above and the first set was planted on 9 March 2020 and the second set 17 days later. As a result of poor establishment, 224 lines of the population were retained for the analysis.

### Statistical analysis

A multiplicative mixed linear model was used for the analysis of traits in each of the three experiments and was implemented in the R package ‘AsReml v 4.1.0.98’ in the R environment (v3.6.3; R Core Team 2021). The best spatial model was fitted using the method described in Isik *et al.* (2017). Generalised heritability for each trait at each experiment was calculated following the generalised fomula for unbalanced data in Cullis *et al.* (2006). Best linear unbiased predictions (BLUPs) which can also be referred to as the genetic value (GV) were obtained from the model.

Correlations were determined with the GVs. Principle component analysis by singular value decomposition using the scaled and centred GVs was conducted. The vectors (traits) and values (lines) for the first two principle components were displayed using a biplot (Kroonenberg 1997).

### Genome-wide association

As the populations had been exposed to a level of selection or derived from multiple F1 and crosses, they were unsuitable for composite interval mapping and hence a genome-wide association approach was undertaken. Separate association analyses were performed on each trait using BLUPs obtained from the models described above for the KKN, YC12147, YC12157, and multi-background populations. A compressed linear mixed model approach (Yu *et al.* 2006) was implemented in the R software package ‘rrblup’ (Endelman 2011). A leave-one-approach was adopted as described by Yang *et al.* (2014) and a clustering approach was used to select the markers used in the kinship matrix with only uncorrelated markers selected (r^2^ > 0.25) on each chromosome (Wang *et al.* 2011). The kinship matrix was calculated as per VanRaden (2008). The function was modified to extract marker effects, and estimated additive effects were given as the peak marker, where a positive effect for all traits indicates Norin PL8 is the donor.

The genomic inflation value, λ, was used to determine the appropriate fit of model (Devlin and Roeder 1999). It was determined that no experimentation required the incorporation of covariates to correct for population structure. Significance thresholds were determined using the “simpleΜ” method as described by Gao *et al.* (2008) using “cor” and “eigen” functions in base R with the number independent test being taken as the number vectors required to explain 99.9% of the variation. Thresholds for Experiment 1, Experiment 2 YC12147, Experiment 2 YC12157 and Experiment 3 were 0.0019, 0.0005, 0.0002 and 0.00012, respectively. Significant SNPs within 50% linkage disequilibrium was classified as a single QTL and named according to McCouch and Committee on Gene Symbolization Nomenclature and Linkge Rice Genetics Cooperative (2008) using the standardised trait name “YMCT” and “YMCTF” for young microspore cold tolerance for spikelet fertility and floral traits, respectively.

## Results

### Experiment: 1 KKN Population

In the KKN population, spikelet fertility was a highly heritable trait (0.81, Table 1). The tolerant, parent Norin PL8 had a high GV for spikelet fertility (86%), while Kyeema had a moderately low GV (44%). A number of lines from the KKN population displayed a lower GV than Kyeema, however, none were higher than Norin PL8.

**Fig. 1.**
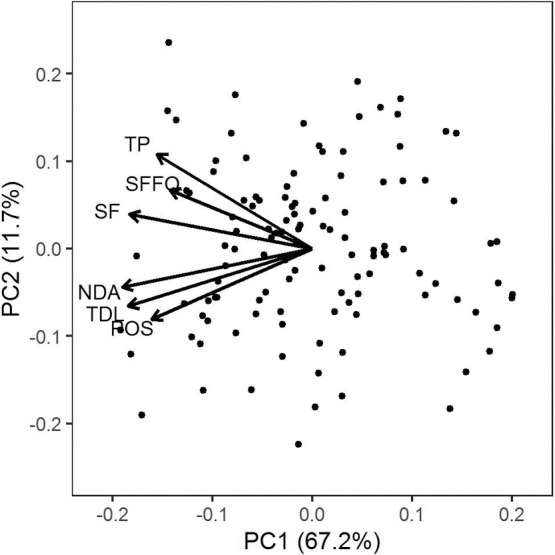
Biplot of the first two component of principle component analysis of spikelet fertility (SF), total pollen in anther (TP), total dehiscence length (TDL), number of dehisced anthers (NDA), pollen on stigma (POS) and the number of tolerant alleles for spikelet fertility and floral traits (SFFQ) in Experiment 1.

For the four floral traits examined, Norin PL8 had higher GV than Kyeema. The traits were moderately to highly heritable. Converse to spikelet fertility, transgressive segregation was observed on both sides of the distributions with the mean GV for top 10% equalling or exceeding the performance of Norin PL8. In the principal component analysis of spikelet sterility, the first two components explained 78.9% of the variance for floral traits and the number of tolerant alleles for fertility and floral traits (discussed below). It reaffirms the high positive correlations between floral traits and spikelet fertility (0.54** to 0.77**; Fig. 1). The phenotypic data was described in detail by Susanti *et al.* (2019).

**Fig. 2.**
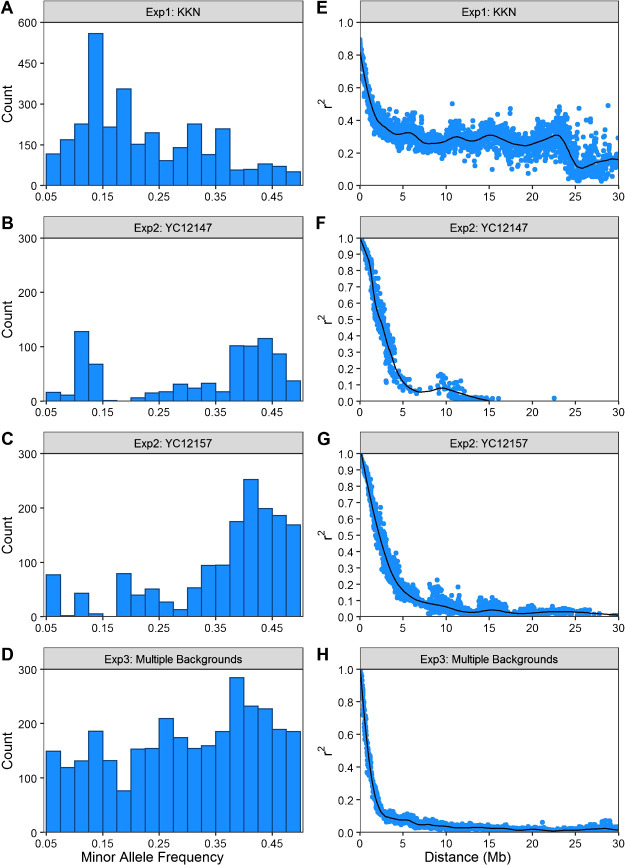
Minor allele frequency (a–d) and r^2^ plotted against physical distance with a linkage disequilibrium decay curve fitted (e–h) for the four populations evaluated from the three experiments for the final filtered marker set. a, e: KKN populations in Experiment 1; b, f: YC12147 in Experiment 2; c, g: YC12157 in Experiment 2; and d, h: multiple background in Experiment 3.

After filtering markers for minor allele frequency, call rate and duplicates, there were 3083 high-quality polymorphic markers from an original 23078 markers. Chromosome 9 had the least number of markers with 97, while chromosome 11 had the most with 551. The median distance between markers was 117 kb with a first and third quartile being 10 kb and 198 kb. There was a large proportion of relatively rare alleles with 1639 SNPs with a minor allele frequency lower than 0.2 (Fig. 2A). There was a high level of linkage disequilibrium in the population with the average r^2^ not falling below 0.5 until 1.55 Mb and 0.3 at 6.05 Mb (Fig. 2E). Linkage disequilibrium remained relatively static at 0.3 until 23 Mb.

Five genomic regions were identified to be significantly associated with spikelet fertility with all but one comprising of one SNP (Table 2). The QTL *qYMCT10.1* on the short-arm of chromosome 10 consisted of two SNPs with an estimated additive effect of 13%. The other four regions were identified on chromosome 1, 4, 5 and 11 with an estimated additive effect of 11–17%. Norin PL8 was determined to be the donor of tolerance for *qYMCT5.1*, *10.1* and *11.1*, we cannot confidently declare a donor for *qYMCT1.2* and *4.1* due to the genotyped parents being called as the same allele.

Seven genomic regions were identified to be associated with floral traits, *qYMCTF1.2* and *qYMCTF10.1* were co-locating with spikelet fertility QTL *qYMCT1.1* and *qYMCT10.1*, respectively. In addition to spikelet fertility, *qYMCTF1.2* was found to be associated with total pollen in anther and the number of dehisced anthers, whereas *qYMCTF10.1* was associated with total dehiscence length and the number of dehisced anthers. The remainder of the floral trait QTL were located on chromosome 1, 2, 5 and 8 (2), and apart from *qYMCTF1.1* were each only associated with one floral trait. Like *qYMCTF10.1*, *qYMCTF1.1* was associated with the total dehiscence length and the number of dehisced anthers. The two QTL on chromosome 8 were both associated with pollen on stigma, given the linkage disequilibrium they could potentially be the one QTL. The *qYMCTF2.1* and *qYMCTF5.1* were associated with total dehiscence length and total pollen in anther, respectively. In the principal component analysis of spikelet fertility, the first two components explained 78.9% of the variance for floral traits and the number of tolerant alleles for fertility and floral traits. It reaffirms the high positive correlations between floral traits, and spikelet fertility. Additionally, the principal component analysis demonstrated that the accumulation of favourable alleles, whether for spikelet fertility or floral traits, contributes to a general increase in spikelet fertility.

### Experiment 2: Double backcrossed populations

The phenotyping of Experiment 2 had a lower heritability (0.45) than Experiment 1 which is expected given the use of double backcrossed populations (Table 1). Norin PL8 had the highest GV for spikelet fertility (80%) while Kyeema had a moderate GV (59%).

The YC12147 and YC12157 populations had 809 and 1560 SNPs, respectively after marker filtering with 9423 markers prior to filtering. YC12147 had one SNP located on chromosome 12 with the remaining chromosomes having between 4 and 141 markers on each chromosome. YC12157 had between 24 and 261 SNPS on each chromosome. The median marker distance was 40 and 36 kb for YC12147 and YC12157, respectively. There were clear segments that were fixed for the Kyeema background in both populations. Linkage disequilibrium decay was similar in both populations with average 0.5 decay occurring after 2.25–2.34 Mb and 20% at 4.16–4.39 Mb (Fig. 2B, 2C, 2F, 2G).

No significant QTL regions were identified in YC12147. One QTL consisting of five markers was identified in YC12157 on the short-arm of chromosome 6, *qYMCT6.1*. Further investigation revealed three haplotypes within the population for the five markers: all homozygous for the Kyeema (HAP1), all homozygous for the NorinPL8 allele (HAP2), and the first SNP homozygous for the Kyeema allele, while the remainder consisted of the Norin PL8 allele which contained 13, 93 and 4 lines, respectively (Tables 2, 3). HAP1 had the highest spikelet fertility (47%) and HAP2 the lowest (33%). HAP3 was between HAP1 and HAP2, while there were four individuals which had a mean spikelet fertility of 38% which was highly suggestive the QTL was located between 5262749–5547900 bp on chromosome 6.

### Experiment 3: Multiple backgrounds

**Fig. 3.**
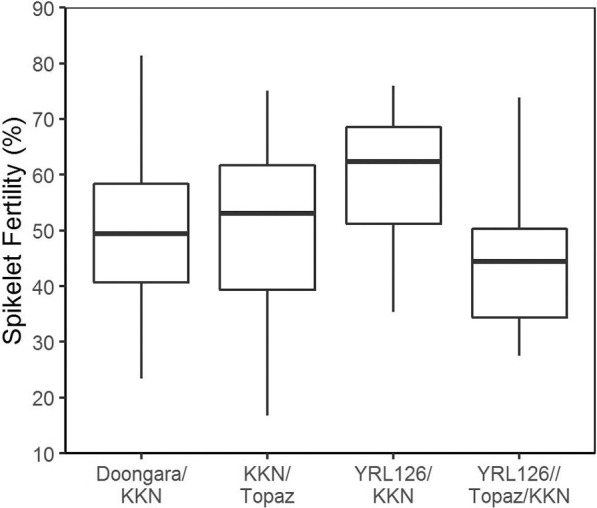
The distribution of spikelet fertility GVs of the four main families evaluated in Experiment 3. The heavy black line shows the median, upper and lower edges of boxes shows the upper and lower quantiles, and whiskers the range.

The phenotyping in Experiment 3 had a high heritability of 0.71. GV for spikelet fertility ranged from 17–82% (Fig. 3). On a pedigree basis, YRL126//Topaz/KKN was considered more susceptible while YRL126/KKN more tolerant than Doongara/KKN and KKN/Topaz, however, the most susceptible and tolerant individuals were present in KKN/Topaz and Doongara/KKN, respectively.

16085 markers were filtered to 3098 high quality SNPs with a median distance of 28.6 kb. There was a large region on chromosome 1 that had no coverage (11.4 Mb) as a result of the region being homozygous. The largest interval markers, excluding chromosome 1, was 5 Mb on chromosome 12. Average linkage disequilibrium decay reached 0.5 at 0.93 Mb and 0.2 at 1.87 Mb (Fig. 2D, 2H). A QTL was identified on the long-arm on chromosome 9, *qYMCT9.1*, with an estimated additive effect of 8% (Table 2). The QTL on the long-arm chromosome 9 consisted of 4 SNPs in 58,500 bp region between 15076724 and 15135224.

### QTL in common

There was no QTL in common identified across the three experiments (Table 4). In Experiment 1, all the SNPs associated with QTL identified with the exception of *qYMCTF1.1* and *qYMCTF1.2/qYMCT1.1* were monomorphic. The SNPs associated with *qYMCTF1.1* and *qYMCTF1.2* were polymorphic in populations used in Experiments 2 and 3. The QTL *qYMCT6.1* was identified in the YC12157 population, the region was polymorphic in Experiment 3 with the peak marker identified in Experiment 2 being at a comparable allele frequency in Experiment 3, but the tolerant haplotype identified in Experiment 2 had a frequency below 0.05. The peak marker for *qYMCT6.1* was not present in the finalised genotype data in Experiment 1, however, a flanking marker (5977699) was present at a low allele frequency (0.10). The QTL identified on chromosome 9 in YRL126//Topaz/KKN, YRL126/KKN and Doongara/KKN in Experiment 3 were monomorphic in Experiments 1 and 2.

## Discussion

### QTL for spikelet fertility

In total, seven genomic regions were found to be associated with spikelet fertility after low temperature exposure at YMS across the three experiments. The genomic region *qYMCT6.1* was identified in YC12157 and has not been previously reported in the literature. Haplotype analysis of the YC12157 revealed that the QTL is most likely in 246 kb region between 5.26 and 5.55 Mb, however, additional experimentation with purposely created fine-mapping population are currently being created for confirmation. The donor for *qYMCT6.1* was Kyeema which is not unexpected, as it is known to carry some intrinsic low temperature tolerance at YMS as exemplified by the performance relative to the progeny in Experiments 1 and 2. The region was not identified in Experiment 3, though it was polymorphic, and demonstrated that the tolerant haplotype was at an extremely low frequency (0.05) in the population evaluated which would have hindered its detection. A closer inspection of *qYMCT6.1* using the MSU Osa1 Rice Gene Models database (Ouyang *et al.* 2006) revealed 37 genes in the regions. Of the 37, 15 were conserved hypothetical proteins and genes or non-protein coding transcripts. Of the remaining 22, 8 are considered priori candidate genes which were either enriched genes related to abiotic stimuli or encoding transcription factors which are associated endogenous stimuli.

The QTL *qYMCT10.1* and *qYMCT4.1* were identified on the short-arm and long-arm of chromosome 10 and 4, respectively, in Experiment 1. The SNP associated with *qYMCT10.1* is co-located with previously cloned gene, *qPSR10* (*LOC_Os10g34840*), with the SNP identified here only 60 kb from the functional polymorphism conferring tolerance (Xiao *et al.* 2018). The result is highly suggestive the NorinPL8 carries the tolerant allele for *qPSR10*, and marker development targeting the functional polymorphism and subsequent genotyping of the parents is underway to confirm whether the gene is conferring tolerance in the population. Similarly, *qYMCT4.1* is co-locating with preeminent QTL *Ctb-1* and *Ctb-2* identified by Saito *et al.* (2001) with the SNP located between the two and Norin PL8 is a known donor. However, it is important to note that the parents were called as monomorphic for *qYMCT4.1*. These results suggest that *qPSR10*, *Ctb-1* and *Ctb-2* are valuable QTL to target with MAS in the Australian breeding program.

The region, qYMCT1.1/*qYMCTF1.2*, detected in the KKN population is likely be an artefact of the selection rather than a QTL conferring low temperature tolerance as the region was polymorphic in all other populations and not detected. Additionally, the favourable allele in the KKN population had an high allele frequency (0.85). The last remaining QTL identified in Experiment 1, *qYMCT5.1* and *qYMCT11.1*, were monomorphic in both Experiments 2 and 3. Both regions have previously been identified in the literature with *qYMCT5.1* co-locating with a region previously identified by Mitchell *et al.* (2016) and Lei *et al.* (2018), and both by Guo *et al.* (2020) which suggest potential value for targeting them both with MAS.

The only QTL within the study that was detected in multiple backgrounds was *qYMCT9.1* within Doongara/KKN, YRL126/KKN and YRL126//Topaz/KKN populations in Experiment 3 demonstrating that it is a prime candidate for MAS for improved low temperature tolerance. Furthermore, the *qYMCT9.1* co-locates with QTL identified in two previous studies providing additional credence for its use in MAS (Guo *et al.* 2020, Lei *et al.* 2018, Suh *et al.* 2010).

### Floral traits and spikelet fertility

The significant correlations between spikelet fertility and floral traits are well-established with higher pollen in anther, number of dehisced anthers and pollen on stigma all contributing to increased spikelet fertility (Farrell *et al.* 2006b, Susanti *et al.* 2019). The biplot of the spikelet fertility and floral traits demonstrated that with increasing the number of tolerant alleles, whether for floral traits or spikelet fertility, that there was a trend for the increase in the GVs for floral traits and spikelet fertility. Additionally, the biplot highlights the highly polygenic nature of inheritance of low temperature tolerance, and the importance of background genetics/the accumulation of small effect QTL for delivering a high level of low temperature tolerance. However, given their small effect size QTL associated with floral traits have limited merit for MAS in the improvement of low temperature tolerance unless also associated with spikelet fertility.

The QTL *qYMCT10.1/qYMCTF10.1* was identified for spikelet fertility and was also demonstrated to be associated with total dehiscence length and the number of dehisced anthers, and highlights the importance of the ability of anthers to dehisce and release viable pollen to ensure successful fertilisation. To the best of our knowledge, this the first time that a QTL has been identified co-locating with both spikelet fertility and the number of dehisced anthers in relation to low temperature exposure during the reproductive stage. Susanti *et al.* (2019) demonstrated the importance of anther dehiscence to increase spikelet fertility and the relative importance of high pollen abundance to drive anther dehiscence. Additionally, *qYMCT1.1*/*qYMCTF1.2* was also associated with total pollen in anther, number dehisced anthers and spikelet fertility further demonstrating the importance of the above, but as described above the region was most likely a result of selection.

Given its relative frequency in the other populations, it can be presumed *qYMCTF1.1* was also the result of selection unless there was no pleiotropic effects between the floral traits and spikelet fertility. The remainder of the four QTL identified for floral traits were monomorphic in the other populations and had no significant association with spikelet fertility which is the target trait.

### Breeding for low temperature tolerance

The QTL that appear to have merit in MAS must firstly be validated in multiple backgrounds to demonstrate their utilitarian value as well as the development of appropriate markers (Cobb *et al.* 2019). The poor utilitarian value of QTL has previously been shown with Fukushima *et al.* (2017) demonstrating no benefit of *Ctb-1* in BC2 of Fukuhibiki (recurrent) and Hokkai IL3. Thus, for *qYMCT5.1*, *6.1*, *9.1* and *11.1* to effectively be deployed, they first must be validated in several backgrounds and suitable high-throughput markers to target the QTL need to be developed. Whereas *qYMCT10.1* and *qYMCT4.1* need to be shown to be synonymous with q*PSR10* and, *Ctb-1* and *Ctb-2*, respectively. If shown to be synonymous, they could be deployed immediately in MAS with the appropriate markers as their utilitarian value has previously been demonstrated (Guo *et al.* 2020, Xiao *et al.* 2018). Though it would be wise for further evaluation which can be achieved with a half-sib crossing program concurrent with introgressing the QTL in an improved background.

The overall breeding aim was to generate a range of long-grain types with low temperature tolerance introgressed and utilise material with three distinctive cycles which was evaluated across three experiments. High fertility individuals were identified across all experiments even though some populations had a single QTL identified. The high fertility firstly demonstrates the polygenic and complex inheritance for low temperature tolerance at YMS but also the perils of advancing material with no prior selection. The perils of no prior selection in cross selection can be seen with *qYMCT10.1* and *qYMCT4.1* which was identified in Experiment 1 (KKN population), however was monomorphic in both Experiments 2 and 3. A crossing program to introgress it into elite long grains is underway which will be used to validate marker development and its benefit to the Australian breeding program, and explore the benefits of stacking identified QTL.

We aimed to identify QTL associated with low temperature tolerance at YMS that has relevance to the Australian breeding program for potential deployment in MAS. Six genomic regions were identified associated with spikelet fertility and which have merit in MAS. A QTL, *qYMCT10.1/qYMCTF10.1*, was associated with spikelet fertility and the number of dehisced anthers which demonstrates the link between spikelet fertility and male reproductive morphology contributing to low temperature tolerance. All but one genomic region was co-locating with QTL previously identified in the literature of which two were cloned. The QTL *qYMCT6.1* has not been previously identified, nor have the genetic association between spikelet fertility and the number of dehisced anthers. Additional validation is necessary before their routine implementation in a breeding program. Additionally, the KKN population demonstrated the potential perils of using selected populations in association analysis. Further research is needed to validate the benefit of identified QTL to the breeding program, develop the appropriate markers for deployment to select for the QTL and explore the benefits of stacking multiple QTL together. The findings of the current study have implications for breeding for low temperature tolerance in the Australian rice breeding program as well as other temperate breeding programs around the world.

## Author Contribution Statement

All authors contributed to the study design. J.M, S.F, C.P, B.C and I.G conceived and guided the experimentation conducted. P.S and B.O generated the populations used in the studies. Z.S and C.P carried out the experimentation. C.P and B.C performed the data analysis. The first draft of the manuscript was written by C.P.

## Figures and Tables

**Table 1.** Summary of genetic values for spikelet fertility and floral traits in Experiment 1 and spikelet fertility in Experiment 2. Top 10% and bottom 10% refers to mean of top and bottom, 10% respectively for each respective trait

	Experiment 1					Experiment 2	
	Spikelet fertility (%)	Total pollen in anther	Total dehiscence length	Number of dehisced anthers	Pollen on stigma	YC12147 spikelet fertility (%)	YC12157 spikelet fertility (%)
Skewness	0.16	–0.42	–0.26	–0.41	1.29	–0.24	–0.59
Kurtosis	2.02	2.67	2.44	2.12	4.79	2.5	2.69
Mean	42	997	604	3.5	48	47	41
Minimum	8	217	195	1	22	25	25
Maximum	77	1707	1015	5.2	125	72	71
Top 10%	71	1501	921	5.1	97	68	63
Bottom 10%	11	331	235	1.2	23	30	23
Kyeema	44	677	440	2.4	24	59	
Norin PL8	86	1506	775	5.1	50	80	
Heritability	0.81	0.76	0.68	0.70	0.51	0.45	

**Table 2.** Significant regions (p-value) associated with spikelet fertility and floral traits identified in the three experiments. Estimated additive effect of the peak marker are in parenthesis with the associated trait. The number of significant SNPs associated with the QTL is in parenthesis in position column

Experiment	QTL	Chromosome	Position	Spikelet fertility (%)	Total pollen in anther (pollen/anther)	Total dehiscence length (μm)	Number of dehisced anthers (anthers/spikelet)	Total pollen on stigma (pollen/spikelet)
Exp 1: KKN	*qYMCTF1.1*	1	2375513 (2)			0.0002 (–96)		
	*qYMCTF1.1*	1	2772610 (3)				0.0003 (–0.7)	
	*qYMCT1.1/qYMCTF1.2*	1	32799035 (1)	0.0012 (–14)	1.66 × 10^–4^ (–273)		0.0013 (–0.9)	
	*qYMCTF2.1*	2	980958 (1)			0.0009 (105)		
	*qYMCT4.1*	4	31113337 (1)	0.001 (17)				
	*qYMCT5.1*	5	5640496 (1)	0.001 (11)				
	*qYMCTF5.1*	5	19142580 (1)		0.001 (263)			
	*qYMCTF8.1*	8	15986648 (2)					0.0007 (–14)
	*qYMCTF8.2*	8	20150360 (1)					0.0009 (16)
	*qYMCT10.1/qYMCTF10.1*	10	18532257 (2)	6.17 × 10^–5^ (13)		0.0003 (130)	5.75 × 10^–5^ (0.88)	
	*qYMCT11.1*	11	7106732 (1)	0.0007 (12)				
Exp 2: YC21157	*qYMCT6.1*	6	5262749 (5)	0.0002 (–8)				
Exp 3: Multiple background	*qYMCT9.1*	9	15081259 (4)	0.0001 (8)				

**Table 3.** Haplotypes identified on the region chromosome 6 in YC12157 with K and N being homozygous for the Kyeema and Norin PL8 allele, respectively

Haplotype	Number of lines	Marker					Mean spikelet fertility*^a^*
		5262749	5547900	5556514	5977699	6194405	
HAP1	13	K	K	K	K	K	47 a
HAP2	93	N	N	N	N	N	33 b
HAP3	4	K	N	N	N	N	39 ab

*^a^* Means followed by the same letter are not significant at p = 0.01.

**Table 4.** Minor allele frequency of peak SNPs for QTL associated with spikelet fertility and floral traits across the 3 experiments. Bold indicates that the QTL was identified within the populations and underline depicts that it was significant for a floral trait and not spikelet fertility

	Chromosome	Position	Exp 1: KKN	Exp2: YC12147	Exp2: YC12157	Exp3: All
*qYMCTF1.1*	1	2375513	**0.48**	0.46	0.48	0.45
*qYMCTF1.1*	1	2772610	**0.50**	0.47	0.49	0.45
*qYMCT1.1/qYMCTF1.2*	1	32799035	**0.15**	–	**–**	0.33
*qYMCTF2.1*	2	980958	**0.11**	–	–	0.39
*qYMCT4.1*	4	31113337	**0.06**	–	–	–
*qYMCT5.1*	5	5640496	**0.15**	–	–	–
*qYMCTF5.1*	5	19142580	**0.15**	–	–	0.45
*qYMCT6.1*	6	5262749	–	–	**0.14**	0.16
*qYMCTF8.1*	8	15986648	**0.12**	–	–	0.07
*qYMCTF8.2*	8	20150360	**0.13**	–	–	–
*qYMCT9.1*	9	15081259	–	–	–	**0.15**
*qYMCT10.1/qYMCTF10.1*	10	18532257	**0.23**	–	–	–
*qYMCT11.1*	11	7106732	**0.32**	–	–	–
